# Family Psycho-Social Involvement Intervention for severe mental illness in Uganda

**DOI:** 10.4102/sajpsychiatry.v30i0.2138

**Published:** 2024-01-30

**Authors:** Racheal Alinaitwe, Musisi Seggane, Andrew Turiho, Victoria Bird, Stefan Priebe, Nelson Sewankambo

**Affiliations:** 1 Department of Psychiatry, College of Health Sciences, Makerere University, Kampala, Uganda; 2 Unit for Social and Community Psychiatry, Queen Mary University of London, London, United Kingdom; 3 Department of Medicine, College of Health Sciences, Makerere University, Kampala, Uganda

**Keywords:** severe mental illness, family psycho-social support intervention, resource-oriented, efficacy, Uganda

## Abstract

**Background:**

Treatment rates for severe mental illness (SMI) are low in low- and middle-income countries because of limited resources. Enlisting family support could be effective and low cost in improving patient outcomes.

**Aim:**

The article assess the feasibility, acceptability and estimates of efficacy of Family Psychosocial Involvement Intervention (FAPII) for patients with SMI.

**Setting:**

Masaka Regional Referral Hospital and Mityana District Hospital in Uganda.

**Methods:**

This was a controlled pilot study with two sites randomly assigned as intervention and control. Thirty patients each with one or two family members and six mental health professionals were recruited at the intervention site. Five patients, their family members and two mental health professionals met monthly for 6 months to discuss pre-agreed mental health topics. Patient outcomes were assessed at baseline, 6- and 12-months and analysed using paired t-tests. The trial was prospectively registered (ISRCTN25146122).

**Results:**

At 6 and 12 months, there was significant improvement in the QoL in the intervention group compared to the control (*p* = 0.001). There was significant symptom reduction in the intervention group at 6 and 12 months (*p* < 0.001). Family Psychosocial Involvement Intervention affected better treatment adherence at 6 and 12 months (*p* = 0.035 and *p* < 0.001, respectively) compared to the control arm.

**Conclusion:**

Family Psychosocial Involvement Intervention improved QoL, medication adherence, reduced stigma and symptoms among patients with SMI. The authors recommend involving families in the care of patients with SMI in Uganda, with FAPII employing culturally sensitive psychotherapy.

**Contribution:**

The results support involvement of family in the care of patients with SMI.

## Introduction

Severe mental illness (SMI) presents a major burden to societies with substantial distress to affected individuals, families and communities resulting in high social, human and economic costs.^1,2^ Severe mental illness is chronic mental illness that affects the quality of life of the individuals, their caretakers and their community.^3^ Mental, neurological and substance use (MNS) disorders contribute 14% of the disease burden globally, accounting for 32.4% of years lived with disability.^3^ The lifetime prevalence of mental illnesses in the Ugandan population is estimated at 35% compared to 13% worldwide, with the prevalence of SMI itself being 3%.^4^

Despite the substantial burden of SMI, treatment rates are low in low- and middle-income countries^5^ including Uganda, where 76% to 85% of people with SMI receive no treatment.^4^ In Uganda, there is only an estimated 1.13 mental health personnel per 100 000 population, and this number includes all mental health cadres.^6^ Further, Uganda’s mental health sector receives only 1% of the meagre health sector budget, making it almost impossible to adequately manage SMI.^7^ This lack of human and financial resources results in late presentation of patients, long clinic queues, high clinician-patient ratios, long hospital stays, lost income and productivity, increased morbidity and mortality and poor prognosis.^1,6^

As a result, there have been calls to implement low-cost resource-oriented interventions in low-income countries to address the mental health treatment gap.^8,9^ There is evidence that resource-oriented approaches like enlisting the support of family members and friends of patients improve the management of SMI and empower individuals.^10^ Models for involving families in the care of patients with SMI, often focusing on psycho-education and family therapy.^11^ The evidence suggests that these approaches can be effective in improving patient outcomes, preventing relapse, increasing medication compliance, reducing hospital re-admissions and shortening stays in hospital.^12,13^ A review of Cochrane Schizophrenia Group Trials Register by Pharoah et al. revealed that family support interventions have been effective in improving the mental health of persons with SMIs like schizophrenia.^13^ However, no research has been conducted in Uganda to investigate the estimates of the efficacy of family psychosocial involvement in the management of SMI.

Psychosocial interventions are modes of treatment other than medication that are used to help individuals with mental health challenges to establish the psychological, social, emotional and intellectual skills to enable them to survive, work and learn within their community independently.^14^ Family has been shown to be a valuable resource in providing psychosocial support for persons with psychosis.^11^ In this study, we aimed to test the estimates of efficacy of the Family Psychosocial Involvement Intervention (FAPII) for patients with SMI in Uganda.

## Research methods and design

### Study design

This was a controlled pilot study that compared FAPII with standard care. The standard care comprised reviews of patients by the clinicians and mostly focused on any signs of relapses, medication side effects and refill of the medication. The intervention group received FAPII in addition to the standard care.

### Study setting

The study took place at two sites in Uganda: Masaka Regional Referral Hospital and Mityana District Hospital. These two sites of Masaka and Mityana were chosen because of their similarity. Both have urban, semi-urban and rural populations are situated on major highways out of Kampala, Uganda’s capital city, are located within the Central region of Uganda and are predominantly inhabited by the same Luganda-speaking agriculturally based cultural group.

Masaka Hospital has a 330-bed capacity and offers both inpatient and outpatient mental health services headed by a principal psychiatric clinical officer. Mityana Hospital is a district hospital with a 100-bed capacity. It offers both inpatient and outpatient mental health services headed by a Psychiatric Clinical Officer (psychiatrist’s assistant). Before any data collection, by flip of a coin, the Masaka Hospital site was randomly assigned the intervention site and the Mityana Hospital the control site.

### Study population

Study participants included consecutively identified outpatients with diagnosed SMI coupled with their caretaker (family members or friend) and their mental health professionals.

### Selection of study participants

At both sites, participants for the study were consecutively recruited from the outpatient mental clinics as it was felt that outpatients would be residing in their communities and also well enough to participate in the intervention. The inclusion and exclusion criteria were:

- Patients with a primary diagnosis of SMI (schizophrenia, bipolar disorder, psychotic or severe depression, epilepsy) assessed by the mental health practitioners using the International Classification of Diseases 10th Revision (ICD-10); aged 18 years to 65; ≥ 6 months in care; had capacity to provide informed consent based on a University of California, San Diego, Brief Assessment of Capacity to Consent (UBACC)^15^ score of ≥ 14; able to communicate in Luganda or English and identified their caregiver, low quality of life assessed by the Manchester Short Assessment of Quality of Life (MANSA score of ≤ 5). We excluded patients who had an organic psychosis or neurocognitive disorder or were inpatients at the time of recruitment. We included epilepsy because this is a frequently encountered neuropsychiatric condition in the outpatient mental health clinics in Uganda, where it presents as an SMI.
- The caretaker was a family member or friend living with the patient in one household, aged 18 years or older. We excluded caretakers who did not have contact with the patient or who had plans to move out of the area within the next 6 months.
- The mental health professional was a psychiatric clinical officer (psychiatric assistant), nurse, social worker or counsellor who was currently working at the outpatient clinic in the randomised area for family involvement and had no plans to leave the post within the next 6 months. We excluded mental health professionals who did not have clinical contact with the patients.

The sample size for the intervention arm was 30 outpatients, 60 family members and 6 mental health professionals. The sample size for the controls was 30 outpatients. Consecutive sampling was used at both sites.

### Materials

#### Socio-demographic questionnaire

This was administered to patients and family members only at baseline to collect socio-demographic data.

#### Outcome tools and measures

All outcome measures were collected from participants at baseline, 6- and 12-month follow-up.

**Primary outcome:** This was quality of life (QoL), which was used to determine preliminary estimates of efficacy in the SMI respondents. The *MANSA* was used to assess QoL.^16^ It is a sixteen-item scale in which participants rated their satisfaction across different life domains on a scale ranging from 1 (could not be worse) to 7 (could not be better).

**Secondary outcomes:** The *Objective Social Outcome Index (SIX)*^17^ was used to measure the objective indicators of social outcomes (employment, accommodation and social relationships) for the respondents. Scores range from 0 to 6, with a higher score indicating better social outcomes.

*The Brief Psychiatric Rating Scale (BPRS)*^18^ was used to measure the SMI symptoms of anxiety, depression, mania, hallucinations, delusions and unusual behaviour. Each symptom was rated between 1 and 7.

The *Medication Adherence Rating Scale (MARS)*^19^ was used to assess medication adherence during the preceding week.

The *Internalised Stigma of Mental Illness Inventory (ISMI)*^20^ is a 29-item questionnaire with each item rated on a four-point scale ranging from 1 (strongly disagree) to 4 (strongly agree).

### Study procedures

#### Intervention arm (Masaka site)

The particular form of FAPII used in this study are trialogues which facilitate mutual learning of the clinicians, patients and their family members in regular and open group discussion meetings.^21^ The FAPII^22^ utilises the family as an accessible resource that can be engaged through psycho-education, trialogue and family therapy to support patients with SMI.^11,13,23,24^

At the intervention site, six mental health professionals were recruited, who then screened their clinic caseload with the ICD-10 to diagnose those with SMI. Patients diagnosed with SMI by the clinician on ICD-10 were further screened by research assistants (RAs) for capacity to consent (UBACC ≥ 14) following which, they were consented. Those with the capacity to consent were assessed for their quality of life by the RA using the MANSA and those with a score of ≤ 5 were included in the study. Eligible patients who agreed to participate were asked to provide contact details for up to two family members with whom they would like to attend the trialogue sessions. These family members provided written informed consent and completed baseline assessments. The patients completed baseline assessments covering socio-demographic characteristics, symptom severity, objective social situations, client service use and medication adherence, stigma and quality of life. Then they carried out the FAPII, which involved regular meetings consisting of group trialogues between five patients, their family members and two mental health professionals. During these sessions, participants discussed topics that were co-produced or pre-agreed upon in the group (e.g. medication, stigma). Trialogues were held once per month over 6 months at a local community hall and were facilitated by a patient or family member and overseen by the attending mental health professional. Each session lasted 1 h – 2 h with a snack break. These groups were closed to new participants joining once the intervention had begun. Each pair of mental health professionals facilitated two groups. Mental health professionals received training in group facilitation. Research assistants and site leads provided support when required. At the end of each session, the members of each group agreed on the date, time and topic of the next session and chose amongst themselves the chairperson and secretary for the next session.

At 6 and 12 months, patient participants and family members were invited to follow-up research assessments with RAs using the above-described tools.

#### Control arm (Mityana site)

Mental health professionals in the Mityana outpatient clinic identified eligible patients with SMI using the ICD-10 from their clinic caseloads. These patients were provided with information about the study and those who agreed were screened again by the RA who administered the UBACC forms to assess their capacity to consent before completing the consent process. Those with the capacity to consent were assessed for their quality of life by the RA using the MANSA and those with a score of ≤5 were included in the study following which, they proceeded to do the baseline assessments as for the intervention group described above. The patients then continued to receive their standard care without any involvement from the research team for the next 6 months. These participants were contacted at 6 and 12 months for follow-up assessments.

### Data analysis

Descriptive statistics were generated for socio-demographic data of all participants. To assess for efficacy of the intervention, mean scores in the intervention group with the control group were compared using a random effect model to allow for the clustering of patients within mental health professionals and adjusting for the baseline value of the primary outcome. A statistical analysis plan was developed prior to data analysis, taking in consideration which covariates were to be adjusted for in the model as well as methods for dealing with missing data. Within-group comparisons were calculated using paired t-tests to test differences between 6 months and baseline and between 12 months and baseline measures with 95% confidence intervals.

We thereafter conducted multivariable linear regression to establish estimates of the intervention effect on QoL at 6 and 12 months while adjusting for other potential confounders. The model used the MANSA scores at 6 months as the outcome variable, while the intervention group was the primary exposure. In the model, we controlled for differences in baseline characteristics by adjusting for variables that were statistically different between the intervention and control groups.

### Ethical considerations

An application for full ethical approval was made to the School of Medicine Research Ethics Committee at the Makerere University College of Health Sciences and ethics consent was received on September 19th 2018. The ethics approval number is (#REC REF 2018-096) and the Queen Mary Ethics of Research Committee on October 31st 2018 (QMERC 2018/67). The study protocol was registered with the ISRCTN registry (ISRCTN25146122). Before any data collection began, all eligible participants were assessed for capacity to consent and written informed consent was obtained from all participants. All participant information was regarded confidential and was kept in locked cabinets and password-protected computers only accessible by the research team. All data were pseudonymised to maintain patient confidentiality. Supervision field visits were carried out by senior members of the research teams.

## Results

**FIGURE 1:**
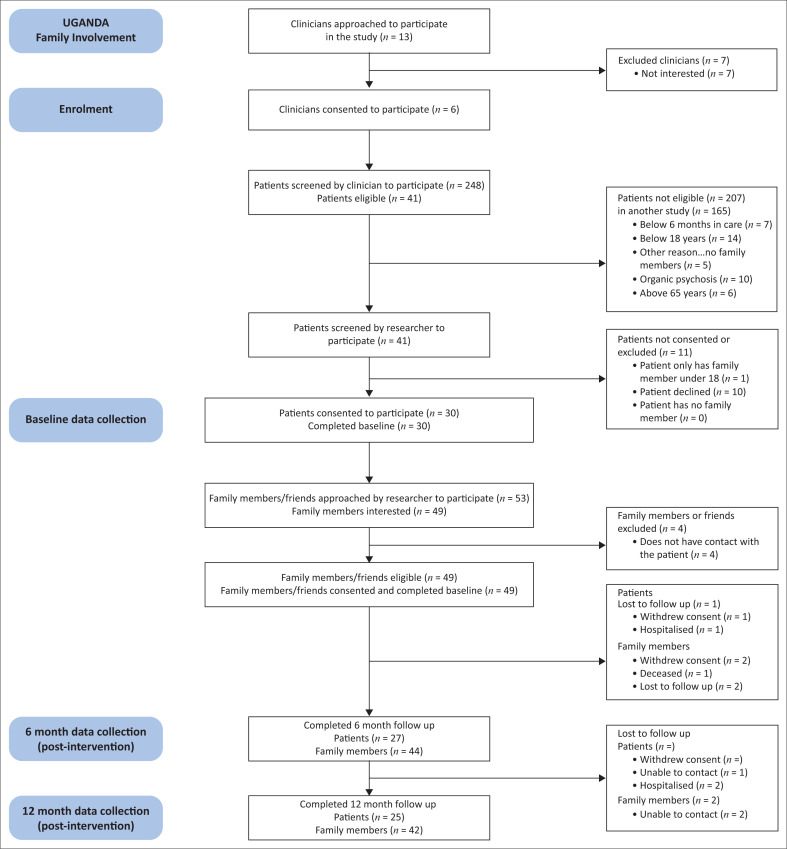
The sampling technique for study participants.

We recruited 30 patients and 6 mental health professionals and 49 family members at the intervention site and 30 patients at the control site. Figure 1 shows a summary of the sampling technique. The socio-demographic characteristics of the SMI patients in both arms are shown in Table 1.

**TABLE 1:** Demographic characteristics of patients.

Characteristics	Intervention group (*N* = 30)				Control group (*N* = 30)				*p*
	*n*	%	Mean	s.d.	*n*	%	Mean	s.d.	
**Gender**									
Female	21	70.0	-	-	20	66.7	-	-	0.781
Male	9	30.0	-	-	10	33.3	-	-	
**Age in years**	-	-	36.7	13.5	-	-	38.2	11.6	0.668
**Marital Status**									
Never married	19	63.3	-	-	9	30.0	-	-	0.020
Married or partnered	8	26.7	-	-	11	36.7	-	-	
Separated or divorced	3	13.3	-	-	10	10.3	-	-	
**Education**									
No formal education	3	10.0	-	-	6	20.0	-	-	0.005
Primary education	5	16.7	-	-	14	46.6	-	-	
Secondary education	18	60.0	-	-	5	16.7	-	-	
Tertiary and/or further education	4	13.3	-	-	5	16.7	-	-	
**Living arrangements**									
Living alone	1	3.3	-	-	3	10.0	-	-	0.217
Living with a partner	16	53.3	-	-	20	66.7	-	-	
Living with friend(s)	13	43.4	-	-	7	23.3	-	-	
**Employment status**									
Employment (part or full time)	10	33.3	-	-	20	66.7	-	-	0.036
Unemployed or voluntary	18	60.0	-	-	9	30.0	-	-	
Student	2	6.7	-	-	1	3.3	-	-	

There were no significant differences in the age distribution, gender composition or living arrangements in both the intervention and control groups. However, the intervention group had more members who were never married compared to the control group (63.3% vs. 30.0%, *p* = 0.020); was more educated (73.3% secondary school and above, vs. 33.4% *p* = 0.005); but more individuals were unemployed (60.0% vs. 30.0% *p* = 0.036).

### Severe mental illness diagnoses and physical health conditions of study participants

The intervention and control groups had similar rates of the SMI diagnoses. Bipolar disorder was the most common disorder in both groups (52.5%) followed by schizophrenia (18.6%) and epilepsy (18.6%). The majority (55.0%) of the respondents had no concomitant physical illnesses. Table 2 summarises these findings.

**TABLE 2:** International Classification of Diseases 10th Revision severe mental illness *diagnoses and physical health conditions of study participants*.

Mental and physical health diagnoses	Intervention		Control		*p*
	*n*	%	*n*	%	
**ICD-10 SMI diagnosis**					
Schizophrenia	5	17.2	6	20.0	-
Bipolar disorder	16	55.2	15	50.0	0.845
Major depressive disorder	3	10.3	2	6.7	-
Epilepsy	5	17.2	6	20.0	-
Dependence syndrome	0	0.0	1	3.3	-
**Physical health conditions** †					
HIV	0	0.0	5	16.7	0.020
Osteoporosis/arthritis	1	3.3	3	10.0	0.301
Dental problem	5	16.7	2	6.7	0.228
Chronic pulmonary disease	0	0.0	1	3.3	0.313
Hypertension/cardiovascular disease	1	3.3	6	20.0	0.044
Obesity	0	0.0	1	3.3	0.301
Diabetes mellitus	1	3.3	3	10.0	0.519
Other physical condition	5	16.7	7	23.3	0.194
None	19	63.3	14	46.7	-

SMI, Severe mental illness; ICD-10, International Classification of Diseases 10th Revision; HIV, human immunodeficiency virus.

† , Self-reported, multiple responses.

### Sessions attendance

The first 6 months of the study were considered the compulsory phase and the next 6 months were optional (flexible phase). The mean number of sessions per patient during the compulsory phase was 5.4 (s.d. 1.6) while that of the flexible phase was 4.2 (s.d. 1.4).

### Outcome measures

#### Primary outcome: Quality of life-Manchester Short Assessment of Quality of Life

QoL scores as measured using the MANSA at baseline, 6 and 12 months are shown in Table 3.

**TABLE 3:** The Effect of FAPII on QoL as measured by the MANSA at Baseline, 6 and 12 months.

Variable	Intervention arm							Control arm						
	Baseline (*N* = 30)		6-month (*N* = 27)		*df*	95% CI	*p*	Baseline (*N* = 30)		6-month (*N* = 29)		*df*	95% CI	*p*
	Mean	s.d.	Mean	s.d.				Mean	s.d.	Mean	s.d.			
MANSA scores: baseline versus 6 months	3.33	0.76	4.27	1.35	0.94	0.4–1.48	0.001	4.10	0.10	4.27	0.77	0.17	−0.14–0.5	0.275
MANSA scores: baseline versus 12 months	3.33	0.76	4.54	0.6	1.17	0.8–0.85	0.001	4.10	0.10	4.41	0.81	0.31	0.10–0.62	0.045

MANSA, Manchester Short Assessment of Quality of Life.

In the intervention arm, there was statistically significant increase in QoL at 6 and 12 months (*p* = 0.001) while in the control arm, there was statistically significant QoL increase only at 12 months (*p* = 0.045).

The results from the multivariable linear regression are presented in Table 4.

**TABLE 4:** Multivariable linear regression assessing the effect of FAPII on QoL as measured by MANSA scores at 6 and 12 months.

Time Period	Variable	β coefficient	95% confidence intervals	*p*-value
6 Months	**Arm**			
	Control	Ref		
	Intervention	0.48	−0.16, 0.13	0.145
	**MANSA score at baseline**	0.19	−0.25, 0.63	0.394
	**Sex**			
	Females	Ref		
	Males	0.40	−0.18, 0.99	0.172
	**Marital status**			
	Single	Ref		
	Married/cohabiting	0.17	−0.55, 0.89	0.645
	Divorced/separated	0.44	−0.35, 1.23	0.274
	**Education level**			
	None	Ref		
	Primary level	1.16	0.35, 1.97	0.005
	Secondary level	1.15	0.34, 1.96	0.006
	Tertiary and above	0.72	−0.30, 1.73	0.165
	**Accommodation**			
	Independent	Ref		
	Supported	−0.5	−1.41, 0.47	0.329
	**Employment**			
	Employed	Ref		
	Unemployed	−0.59	−1.27, 0.08	0.083
	Student	−0.17	−1.41, 1.08	0.791
12 Months	**Arm**			
	Control	Ref		
	Intervention	0.54	0.12, 0.96	0.011
	**MANSA score at baseline**	0.23	−0.05, 0.51	0.11
	**Sex**			
	Females	Ref		
	Males	0.13	−0.25, 0.50	0.506
	**Marital status**			
	Single	Ref		
	Married/cohabiting	0.32	−0.15, 0.79	0.179
	Divorced/separated	0.03	−0.47, 0.54	0.898
	**Education level**			
	None	Ref		
	Primary level	0.36	−0.21, 0.93	0.218
	Secondary level	0.03	−0.58, 0.63	0.929
	Tertiary and above	−0.23	−0.93, 0.47	0.519
	**Accommodation**			
	Independent	Ref		
	Supported	0.16	−0.45, 0.78	0.602
	**Employment**			
	Employed	Ref		
	Unemployed	−0.46	−1.89, -0.02	0.039
	Student	0.15	−0.65, 0.95	0.711

MANSA, Manchester Short Assessment of Quality of Life.

At 6 months, the level of education of the SMI patients remained as the factor significantly affecting QoL. (*p* = 0.005 and *p* = 0.006 for primary and secondary school levels, respectively). The intervention arm had more educated participants. Being in the intervention arm was significantly associated with higher MANSA scores at 12 months (*p* = 0.001)

### Secondary outcomes

The secondary outcome measures are summarised in Table 5.

**TABLE 5:** Secondary Outcomes Measures at Baseline, 6 & 12 Months: SIX, BPRS, MARS & ISMI.

Time Period		Intervention Arm							Control Arm						
		Baseline *N* = 30		6 months *N* = 27		*df*	95% CI	*p*	Baseline *N* = 30		6 months *N* = 29		*df*	95% CI	*p*
	Outcome Scores	Mean	s.d	Mean	s.d				Mean	s.d	Mean	s.d			
6 Months†	SIX	4.41	1.34	4.81	1.18	0.41	−0.21, 1.02	0.184	4.97	1.09	4.79	1.11	−0.17	−0.57, 0.22	0.378
	BPRS	58.53	17.13	39.71	11.42	−17.74	−24.24,-1.24	< 0.001	30.2	4.66	37.52	7.74	7.62	4.59, 10.65	< 0.001
	MARS	5.57	1.50	6.48	2.37	0.90	0.07, 1.72	0.035	6.83	1.44	7.76	1.38	0.9	0.28, 1.51	0.006
	ISMI	22.57	3.6	15.59	4.92	−7.00	−9.38, -4.62	< 0.001	17.07	4.40	16.96	4.25	0.34	−1.20, 1.27	0.955

	**Secondary Outcome Scores**	**Baseline *N* = 30**		**12 months *N* = 25**		***df***	**95% CI**	***p***	**Baseline *N* = 30**		**12 months *N* = 29**		***df***	**95% CI**	***p***
		Mean	s.d	Mean	s.d				Mean	s.d	Mean	s.d			
12 Months†	SIX	4.39	1.36	4.46	1.21	0.77	−0.62,0.77	0.821	4.97	1.09	5.35	1.11	0.38	−0.11, 0.87	0.126
	BPRS	58.53	17.13	33.31	8.75	−23.77	−29.74,-7.81	< 0.001	30.82	5.65	30.83	5.65	0.93	−1.69, 3.55	0.473
	MARS	5.57	1.50	7.15	1.46	1.54	0.85, 2.23	< 0.001	6.83	1.44	7.31	1.67	0.45	−0.30, 1.2	0.232
	ISMI	22.57	3.6	19.19	2.09	−3.15	−4.78, −1.52	< 0.001	17.07	4.40	14.66	3.19	−2.27	−3.9, -0.65	0.008

Objective Social Outcome Index; BPRS, Brief Psychiatric Rating Scale; MARS, Medication Adherence Rating Scale; ISMI, Internalised Stigma of Mental Illness Inventory.

† , time period.

There was statistically significant improvement in symptom severity, medication adherence and internalised stigma in the intervention group at 6 months and these were sustained at 12 months.

On multivariable linear regression analysis, high MARS scores were a significant factor affecting QoL of the SMI respondents at 6 months (*p* < 0.001) and at 12 months (*p* = 0.006). In both arms, the SMI subjects’ QoL was significantly affected by stigma at baseline (*p* = 0.006) 6 months (*p* = 0.050) and 12 months (*p* < 0.001).

At multivariate analysis after controlling for possible confounders and the differences in baseline characteristic of MANSA score for QOL, marital status, employment and education level between the two groups, the changes in symptom severity, medication adherence and internalised stigma in the intervention arm remained statistically significant as shown in Table 6.

**TABLE 6:** Significant findings from multivariable linear regression assessing the effect of FAPII intervention on QOL based on the secondary outcomes scales scores.

Secondary Outcome	Variable	6 Months *N* = 27			12 Months *N* = 25		
		β coefficient	95% CI	*p*	β coefficient	95% CI	*p*
Social Index Outcomes (SIX)	**Arm**						
	Control	Ref	-	-	Ref	-	-
	Intervention	4.67	1.22, 17.89	0.024	0.46	0.12, 1.76	0.258
	**Employment**						
	Employed	Ref	-	-	Ref	-	-
	Unemployed	0.09	0.01, 0.99	0.028	0.44	0.03, 6.45	0.551
	Student	0.43	0.01, 22.3	0.571	4.00	0.26, 60.9	0.318
Brief Psychiatric Rating Scale (BPRS)	**BPRS score at Baseline**	0.22	0.01, 0.43	0.036	0.20	0.03, 0.36	0.018
Medical Adherence Rating Scale (MARS)	**MARS score at baseline**	0.56	0.25, 0.87	< 0.001	0.361	0.103, 0.620	0.006
Internalised Stigma of Mental Illness Inventory	**Arm**						
	Control	Ref	-	-	Ref	-	-
	Intervention	−2.58	−5.17, 0.00	0.050	3.34	1.8, 4.89	< 0.001
	**ISMI score at baseline**	0.39	0.11, 0.68	0.006	0.08	−0.09, 0.25	0.361
(ISMI)	**Education level**						
	None	Ref	-	-	Ref	-	-
	Primary level	4.64	1.34, 7.94	0.006	−2.59	−4.76, -0.41	0.020
	Secondary level	4.28	0.91, 7.65	0.013	−0.42	−2.77, 1.92	0.725
	Tertiary & above	3.94	−0.11, 7.99	0.057	−1.12	−3.74, 1.50	0.403
	**Accommodation**						
	Independent	Ref	-	-	Ref	-	-
	Supported	−4.62	−8.36, -0.87	0.016	−0.43	−2.67, 1.82	0.709
	**Employment**						
	Employed	Ref	-	-	Ref	-	-
	Unemployed	−0.60 (-3.29, 2.10)	-	0.664	2.13	0.54, 3.72	0.009
	Student	−3.83 (-8.93, 1.28)	-	0.142	−1.41	−4.41, 1.60	0.359

SIX, Objective Social Outcome Index; BPRS, Brief Psychiatric Rating Scale; MARS, Medication Adherence Rating Scale; ISMI, Internalised Stigma of Mental Illness Inventory.

## Discussion

This study set out to investigate the estimates of efficacy of FAPII for SMI in the Ugandan context using a controlled pilot study design. It addressed the research question of whether patient outcomes could change when FAPII was used.

The two arms had similar rates of the SMI diagnoses with bipolar disorder being the most common in both groups, followed by schizophrenia, epilepsy and depression in that order. These are the usual SMI diagnoses in hospital-based mental health clinics in Uganda as they tend to cause disruptions in the community.^25,26^ The Intervention group had no HIV patients because these attended a separate specialised clinic at their hospital. Participant attendance was at 83.3% of the sessions.

QoL was the primary outcome of this study as measured by the MANSA scores QoL was generally higher among the more educated and employed. Severe mental illnesses tend to start in the youthful years of affected individuals.^27^ This negatively impacts their schooling and hence stops them from acquiring education and skills training for good future employability. This contributes to lack of critical job skills hence unemployability and contributes to their downward social class drift and the resultant low socio-economic status of these individuals with SMI.^28^ Our finding is in keeping with other studies that have demonstrated that family involvement with its associated shared decision-making (SDM) involving patients improves treatment outcomes and a better QoL.^23,29^ In the Ugandan setting, lack of education, poor vocational skills and unemployment are not usually addressed as common components of psychiatric therapy.^30^ These issues were addressed in the intervention arm trialogue discussions, indicating the importance of FAPII in addressing concerns impacting on the QoL of the SMI respondents, hence calling for Ugandan researchers to innovate for culture and context sensitive psychotherapies in Ugandan settings, such as Group Support Psychotherapy, GSP.^30,31^ Family Psychosocial Involvement Intervention offered an opportunity for patients to jointly discuss matters regarding their conditions with their families and health care providers. This enabled a better understanding of the lived experiences of the SMI patients on the part of their families and health care providers and probably reduced on expressed emotions and critical comments.^32^

There were no significant differences in the social index outcomes as measured by the mean SIX scores in both study arms at 6 and 12 months. This finding is at odds with studies in highly developed countries where social index outcome scores have been reported to be impacted by family involvement.^23,33,34^ Our finding could be accounted for by the traditional social connectedness and family cohesion, which is still present in Ugandan communities whereby family bonds and social networks protect the vulnerable.

As regard to symptoms, we found that at 6 and 12 months, the mean BPRS scores in the intervention arm showed a statistically significant reduction unlike the control arm with statistically significant increase in mean BPRS scores at 6 months. This finding clearly indicates that FAPII positively impacted SMI symptom reduction, similar to the findings of Muhić et al. and Ramon et al., the latter who investigated the use of Family Group Conferencing in adult mental health work.^21,33^ Also, Sikira et al. had similar findings in a Bosnia study, which employed a similar methodology to our study.^23^

The mean MARS scores at 6 months significantly increased for both arms. However, at 12 months, only the intervention arm significantly maintained the improvement. It would seem that FAPII had a positive impact on medication adherence. This finding is in keeping with Muhić et al. in a Bosnia study which also found improvement in treatment adherence in those SMI patients who received family intervention.^33^ On multivariable linear regression, treatment adherence still came out as a factor significantly impacting the QoL. It would seem that the SMI patients took their medications seriously as it was likely to be always emphasised by their therapists and caregivers.

There was a statistically significant reduction in ISMI scores in the intervention arm. Our study showed that FAPII intervention reduced internalised stigma in a sustained manner. However, it took much longer (12 months) to realise any stigma reduction in the control group. Other studies of family involvement when treating SMI patients have had similar findings.^13,23,33^ These studies cited increased patients’ confidence as family involvement enabled them to participate in decisions impacting their treatment and creating a better bond between patients, their family caretakers and clinicians. In a Pakistan study, Saleem et al. found that multi-family groups provided a non-judgmental space that promoted mutually beneficial treatment decision making and learning between patients, family members and treatment providers.^34^ Matthews et al. (2021) studied SDM involving communication, preferences and collaboration of depressed patients with their health providers during their treatment.^35^ They found that patients preferred sharing information and control over the final decisional outcomes and that trust between patients and providers emerged as a critical precondition to effective reduction of stigma.

Introduction of culture and context-sensitive psychotherapy in the FAPII as provided for in Group Support Psychotherapy, which addresses issues of family, community, income and jobs including group support for income-generating activities^30,31^ would allow for addressing the pertinent psychosocial issues that impact the QoL the most in our SMI respondents.

### Limitations

As far as we know, this was the first study to address FAPII in the care of individuals living with SMI in Uganda. However, our sample size was small as the methodological design was one of a controlled pilot study design. A larger randomised clinical trial would be needed to prove the actual effectiveness of FAPII. Lastly, the coronavirus disease 2019 (COVID-19) pandemic caused delays in the study implementation because of lockdowns and other pandemic Standard Operating Procedures including restricting movements of patients, family caretakers and healthcare providers. This unforeseen and unavoidable limitation reduced the number of sessions attended by our respondents, especially in the flexible phase of the study.

## Conclusion

In summary, our study showed that FAPII in the Ugandan context had a significantly positive impact on improving QoL, symptom reduction and internalised stigma reduction of SMI respondents.

Family Psychosocial Involvement Intervention should be instituted in the psychotherapy of SMI patients. Given the high clinician-patient ratios in Uganda, we recommend for the recruitment of psychologists, psychiatric social workers and certified mental health counsellors as essential career cadres in the Ministry of Health as prerequisites to implementing effective psychosocial interventions in Uganda’s mental healthcare.
